# Salient distractors influence information accrual rather than quitting threshold in visual search

**DOI:** 10.3758/s13414-025-03104-8

**Published:** 2025-06-16

**Authors:** Mark W. Becker, Jeff Moher, Derrek T. Montalvo, Andrew Rodriguez

**Affiliations:** 1 Department of Psychology, Michigan State University, East Lansing, MI 48824 USA; 2 Department of Psychology, Connecticut College, New London, CT 06320 USA

**Keywords:** Quitting threshold effect, Visual search, Drift diffusion models

## Abstract

Moher (*Psychological Science*, *31*[1], 31–42, 2020) recently reported that adding a salient distractor (SD) to a visual search display results in more misses and faster target-absent reaction times, a pattern interpreted as a reduction in the quitting threshold; participants searched less of the display before responding target absent. This finding could have implications for real-world searches with distraction. However, in those experiments, the salient distractor shared critical features with the frequent distractors. In two experiments, we expand on this finding by showing that the pattern of results maintains when a salient distractor does not share critical features with the frequent distractors but reverses when it shares features with the target. The pattern of results is consistent with the salient distractor providing a rapid accumulation of evidence towards its associated boundary in a drift diffusion framework—when it shares features with the target there is a burst of evidence accumulation toward the “present” boundary; when it is a distractor there is a burst of evidence toward the “absent” boundary. We believe this account of the SD’s impact provides a more parsimonious account than a quitting threshold account and can better explain when a salient distractor will harm or help target detection.

Many experiments have used the additional singleton paradigm (Theeuwes, 1990, 1991, 1992) to investigate attentional control. Typically, in this method subjects are asked to search for a prespecified target that is defined by shape and to make a two-alternative discrimination about some aspect of that target. For instance, the task might be to find the diamond shape and respond whether a small dot appearing within the diamond is on the left or right side (Gaspelin & Luck, 2018; Gaspelin et al., 2015). While color is irrelevant to that task, on control trials, all stimuli appear in a single color, while in the additional singleton task, one of the distractors appears in a unique color, making it a color singleton. The usual findings from these experiments are that the singleton captures attention via a bottom-up saliency mechanism, thereby causing slower target reaction times (RTs; Geyer et al., 2008; Theeuwes, 1992, 2004, 2010). Although, recently, a number of authors have used the method to investigate attentional suppression—demonstrating that when the feature of the salient distractor is held constant, people can learn to suppress that feature, thereby reducing or sometimes reversing the capture effect (Gaspelin et al., 2017; Müller et al., 2009; Vatterott & Vecera, 2012).

Traditionally, in this additional singleton paradigm, there is a target on every trial, whereas in many other visual search paradigms, the subject is asked to make a target present/absent judgment, and targets appear on some subset of trials—often, 50% (Dent, 2023; Fleck & Mitroff, 2007; Hout et al., 2015). However, Moher (2020) performed an experiment that was akin to an irrelevant singleton task, but where people made target present/absent judgments. In that method, participants were asked to search for the presence or absence of a vertical line in an array of lines that were tilted slightly to the left or right, or were vertical, and the target appeared on 50% of trials. While color was irrelevant to the task, on control trials, all the lines appeared in the same color. On experimental trials, one of the distractor lines appeared as a highly salient singleton—it was a unique color, twice as large as the other stimuli, and onset 100 ms after the other elements in the array, creating large color singleton with a unique onset.

Using this method, Moher (2020) found that the presence of the highly salient singleton produced slower target detections, replicating the slowing found in the traditional additional singleton method. However, here the singleton also increased miss errors (from 8.8% to 12.6% in Experiment 1) in target-present trials and decreased (from 1,451 to 1,380 ms) target-absent RTs. Taken together, this pattern of more missed targets and faster target-absent RTs was taken as an indicator that the distractor was producing lower search-quitting thresholds (Becker et al., 2022; Chun & Wolfe, 1996; Peltier & Becker, 2016; Wolfe & Van Wert, 2010)—in the presence of a salient distractor, people performed a less thorough search of the display before making a target-absent response—the quitting threshold effect (QTE).

The main QTE finding, that a salient distractor produces faster target-absent RTs and more misses, has been replicated in a number of subsequent papers (Lawrence & Pratt, 2022; Lawrence, Paas, et al., 2023a, b; Lui et al., 2024). Many of these papers have manipulated the saliency of the distractor to find the boundary conditions when a distractor would or would not produce the QTE. For instance, reducing the size of the salient distractor so that it matched the size of other stimuli was enough to eliminate the QTE effect (Lawrence & Pratt, 2022). This is somewhat surprising since the distractor was still a color singleton and onset slightly after the rest of the stimuli—thus, it should have been fairly salient. By contrast, an experiment that eliminated the unique onset of the distractor, such that it appeared at the same time as the other elements of the display, found the QTE (Lawrence et al., 2023a, b). Reducing the size of the salient distractor was more impactful on the QTE than eliminating its unique onset. It also appears that the effect occurs across a wide range of salient distractor prevalence rates, with the effect replicating with distractor prevalence rates from 90 to 20% of trials. But the effect appears to be eliminated if the prevalence rate of the salient distractor is dropped to 10% of trials (Lui et al., 2024). Again, this is somewhat surprising because the very rare appearance of the salient distractor should make it even more salient. Thus, it seems like there is a goldilocks zone, where the salient distractor must be salient enough, but not too salient, to produce the QTE. Importantly, most of these papers included some conditions with parameters similar to the original report, and in those cases the QTE replicated.

While clearly there is more work to be done to determine the boundary conditions of when a salient stimulus will or will not produce the QTE, the goal of the current paper was to investigate why the QTE effect occurs when it does. That is, why should the presence of a salient distractor produce more target misses and faster target-absent RTs. When considering this question, we note that in almost all of the experiments that have investigated the QTE, the targets’ defining feature has been orientation, and the displays have only had three orientations—vertical (the target) and two distractor orientations that were slightly off vertical and mirror images of each other. Further, the orientation of the salient distractor always matched the orientation of the common distractors.^1^

It is possible that this relationship between the salient distractor’s orientation and the orientation of the common distractors is critical to the production of the QTE. To examine whether this relationship was critical, in Experiment 1 we performed a conceptual replication of Moher’s (2020) original method, but informed participants that the target was a *blue* vertical line. We also included an additional condition that involved the presentation of a large salient red distractor that was vertical, so the distractor’s orientation matched the orientation of the target rather than the common distractors.

If all salient distractors reduce quitting thresholds, we should find that both of the salient distractor conditions (vertical and oblique) produce the QTE pattern of more misses and faster target-absent RTs. If, however, the effect of the salient distractor depends on the relationship between its orientation and the orientation of the target and/or common distractors, we may find that the QTE pattern of results disappears or even reverses when the distractor’s orientation matches the target’s. Such a finding might provide some insight into the source of the QTE.

## Experiment 1

### Methods

#### Participants

Prior research on the QTE (Lawrence & Pratt, 2022; Moher, 2020) generally reports fairly large effect sizes for the effect of distractor, usually η_p_^2^ > 0.19. Based on those studies, we were conservative and assumed a η_p_^2^ = 0.1 in a G*Power sample size calculation for a within-factors analysis of variance (ANOVA) with a power = 0.95. The required total sample size was estimated to be 25 participants. Being conservative, we increased this to a target sample size of 40. Forty undergraduate students (*M*_age_ = 19.48 years, *SEM* = 0.30, 32 women, seven males, one no response) participated, in person, for course credit. The experimental procedures were approved by Michigan State University’s IRB, and all participants supplied informed consent prior to participating in the experiment. All participants reported normal or corrected-to-normal vision.

#### Displays

**Fig. 1:**
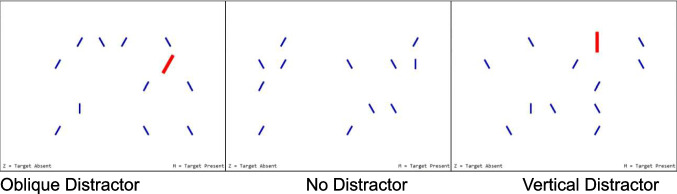
The three distractor conditions in Experiment 1. In the Vertical and Oblique Distractor conditions the salient distractor also onset 100 ms after other stimuli. All three examples are of target present trials, but 50% of trials were target absent, which would be similar to replacing the target with another small blue oblique line

Displays consisted of an array of 12 items (Fig. 1). The target was a vertical blue bar (7 pixels × 47 pixels). The typical distractors were the same blue bar rotated 30° to the left or right of vertical. Three types of salient distractors appeared in red, twice as large as the blue bars, and could be vertical or oblique (rotated 30° to the right or left of vertical). In trials with a salient distractor, the salient distractor appeared 100 ms after the rest of the array so that it produced an isolated abrupt onset. To create the displays, the screen was segmented into a 8 × 5 grid. On a given trial, the 12 stimuli were placed in randomly selected 12 cells from the 40 possible cells.

Each display began with a central fixation cross (500 ms), which was replaced by the search array, which remained visible until responses. Participants pressed the “m” key to indicate the target was present and the “z” key to indicate that the target was absent. To remind subjects of the key mappings, there was “Z = Target Absent” in the lower left corner of the display and “M = Target Present” in the lower right.

#### Procedure

Participants were instructed that the target was a blue vertical bar. The task began with a practice block of 24 trials composed of 12 target-absent displays and 12 target-present displays. Within each set of 12, six were control trials, three included an oblique (distractor match) salient distractor, and three included a vertical (target match) salient distractor. Trial order was randomized.

After the practice block there was a break, followed by a block of 200 trials. The makeup of these trials was similar to the practice block—half of the trials (*n* = 100) were target present and half were target absent (*n* = 100). Within each of those halves, half of the trials (*n* = 50) had a salient distractor, with half (*n* = 25) of the salient distractors being an oblique red distractor (matching the orientation of a typical distractor) and half (*n* = 25) being a vertical red distractor (matching the orientation of the target). Trial order was randomized.

### Results

#### Analyses

An omnibus 2 (target presence: target present/target absent) × 3 (distractor type: oblique, none, vertical) repeated-measures ANOVA was performed for both accuracy and RT. These were followed by separate ANOVAs for the target-present trials and target-absent trials, each with three levels of distractor conditions. For significant ANOVAs, follow-up paired *t* tests were used to localize the effects. RT analyses were run on each subject’s median RT for correct responses in a condition. Data from four subjects were eliminated from further analyses for performing at or below chance in one of the conditions, leaving a sample of 36 on which analyses were performed.

#### Accuracy

**Fig. 2:**
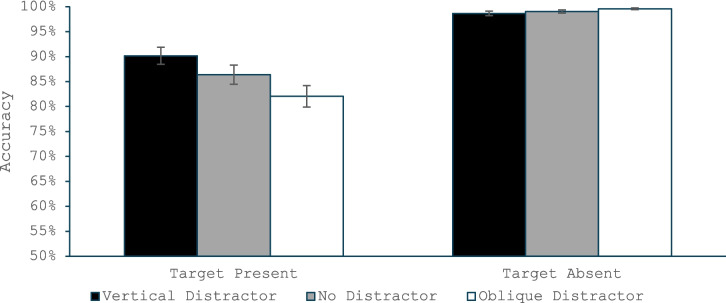
Accuracy in Experiment 1 as a function of whether the trial was a target present or target absent trial and whether the salient distractor matched the target’s orientation, did not appear, or matched the common distractors’ orientation. Error bars are the standard error of the mean

The omnibus ANOVA (Fig. 2) found a main effect of target presence, *F*(1, 35) = 57.46, *p* < 0.001, η_p_^2^ = 0.62, with higher accuracy for the target-absent than target-present trials. There was also a main effect of distractor type, *F*(2, 70) = 9.36, *p* < 0.001, η_p_^2^ = 0.21, and a Target Presence × Distractor Type interaction, *F*(2, 70) = 18.49, *p* < 0.001, η_p_^2^ = 0.35. The ANOVA on only the target-present trials revealed a main effect of distractor type, *F*(2, 70) = 14.57, *p* < 0.001, η_p_^2^ = 0.29, and follow-up *t* test revealed more misses for the oblique-salient distractor than the no-salient distractor condition, *t*(35) = 3.66, *p* < 0.001, *d* = 0.61, while the vertical salient distractor produced fewer misses than the condition with no distractor, *t*(35) = 2.14, *p* = 0.04, *d* = 0.36. Thus, the target-present pattern replicated the typical QTE finding of increased misses, but only when the salient distractor matched the orientation of the common distractors. When its orientation matched the target orientation, the effect on misses reversed. The ANOVA on target-absent trials found no significant difference across distractor types, *F*(2,70) = 1.88, *p* = 0.16, η_p_^2^ = 0.05.

#### Reaction time

**Fig. 3:**
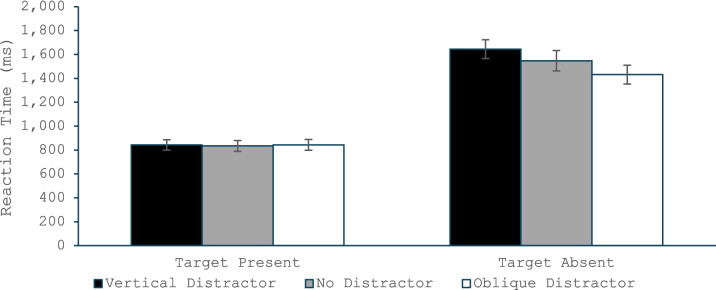
Reaction time in Experiment 1 as a function of whether the trial was a target present or target absent trial and whether the salient distractor matched the target’s orientation, did not appear, or matched the common distractors’ orientation. Error bars are the standard error of the mean

The omnibus ANOVA (Fig. 3) found a main effect of target presence, *F*(1, 35) = 139.84, *p* < 0.001, η_p_^2^ = 0.80, with slower RTs for the target-absent than target-present trials. There was also a main effect of distractor type, *F*(2, 70) = 19.90, *p* < 0.001, η_p_^2^ = 0.36, and a Target Presence × Distractor Type interaction, *F*(2, 70) = 11.99, *p* < 0.001, η_p_^2^ = 0.26. The ANOVA on only the target-present trials revealed no significant effect of distractor type, *F*(2, 70) = 0.06, *p* < 0.94, η_p_^2^ = 0.002. The ANOVA on target-absent trials found a significant difference across distractor types, *F*(2, 70) = 28.62, *p* < 0.001, η_p_^2^ = 0.45, and a follow-up paired *t* test revealed that target-absent responses were made more quickly in the oblique-distractor condition than the no-distractor condition, *t*(35) = 4.20, *p* < 0.001, *d* = 0.70, and were made more slowly in the vertical-distractor than no-distractor condition, *t*(35) = 3.60, *p* < 0.001, *d* = 0.60. Again, these findings replicate the typical QTE effects when the salient distractor matched the orientation of the typical distractor, but the effects reversed when the distractor’s orientation matched the target orientation.

### Discussion

When salient distractors matched the orientation of the common distractors, we replicate the main QTE effect—misses increased and target-absent RTs decreased. However, when the salient distractor matched the orientation of the target, this pattern completely reversed—there were fewer misses and longer target-absent RTs. Clearly, not all salient distractors produce a low quitting threshold, as the relationship between the distractor and the target features matters. In the General Discussion, we will describe our explanation for why these opposite patterns emerged, but before doing so, we report a second experiment, which seeks to replicate the findings of Experiment 1 while adding an additional condition consisting of a distractor that is associated with neither the target’s nor distractor’s orientation. This was done to observe whether the typical QTE result only occurs if the salient feature of the distractor matches the common distractors and/or whether the effect only reverses when the distractor’s orientation matches the target.

## Experiment 2

The methods were similar to Experiment 1, with a few exceptions. First, there was an additional salient-distractor condition in which the salient distractor was a large circle, whose diameter matched the length of the other large salient distractors. Thus, this distractor was large but had no orientation, and while it was a distractor, it matched the orientation of neither the target nor frequent distractors. We also increased the number of trials such that there were 120 target-absent and 120 target-present trails, composed of 30 trials in each of the four distractor (no distractor, oblique distractor, circular distractor, and vertical distractor) conditions. We also thought that holding the color of the salient distractor constant (red) in Experiment 1 might have reduced the size of our effects, since some researchers have found that a constantly colored salient singleton can lead to attention suppression rather than capture (Cunningham & Egeth, 2016; Vatterott & Vecera, 2012). As a result, another change we made in Experiment 2 was that the color of the irrelevant singleton was randomly set to one of four possible colors (red, green, yellow, purple) on each trial. The final difference was that this experiment was run online. Participants were recruited through Prolific, and the experiment was run on E-Prime Go. Given this method of data collection, we anticipated needing to eliminate data from more subjects due to poor performance and that the data might be noisier. Thus, we doubled our projected sample size and recruited 80 participants (*M*_age_ = 41.23, *SEM* = 1.5; 31 women, 47 men, two no response). Using the same criteria as Experiment 1, we eliminated data from any subject who performed at or below chance in any condition. That resulted in the removal of data from 27 participants, leaving a final sample size of 53 participants.^2^

### Results

#### Accuracy

**Fig. 4:**
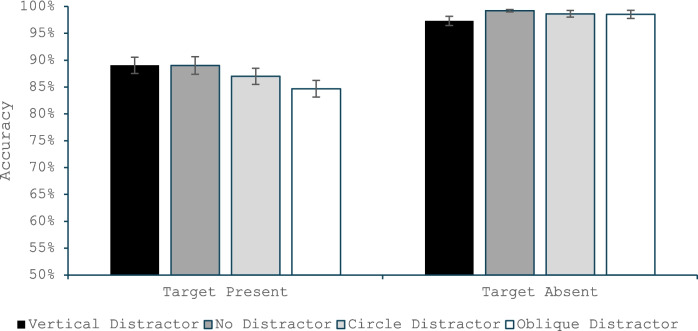
Accuracy in Experiment 2 as a function of whether the trial was a target present or target absent trial and whether the salient distractor matched the target’s orientation, did not appear, was a circle or matched the common distractors’ orientation. Error bars are the standard error of the mean

Like with Experiment 1, the omnibus ANOVA (Fig. 4) found main effects of target presence, *F*(1, 52) = 60.65, *p* < 0.001, η_p_^2^ = 0.54, a main effect of distractor condition, *F*(3, 156) = 7.88, *p* < 0.001, η_p_^2^ = 0.13, and an interaction, *F*(3, 156) = 6.25, *p* < 0.001, η_p_^2^ = 0.11. The ANOVA on only the target present trials revealed a main effect of distractor type, *F*(3, 156) = 8.12, *p* < 0.001, η_p_^2^ = 0.14, and follow-up *t* test revealed that relative to the no-distractor condition, the oblique distractor produced significantly more misses, *t*(52) = 3.68, *p* < 0.001, *d* = 0.51, and the circular distractor also produced marginally more misses, *t*(52) = 1.81, *p* = 0.076, *d* = 0.25. By contrast, the vertical-distractor did not differ from the no-distractor condition, *t*(52) = 0.04, *p* = 0.97, *d* = 0.005. The oblique condition also produced significantly fewer hits than the circular distractor, *t*(52) = 2.38, *p* = 0.021. Unlike Experiment 1, an ANOVA on the target-absent trials found a main effect of distractor condition, *F*(3, 156) = 3.35, *p* = 0.02, η_p_^2^ = 0.06. Follow-up *t* tests reveal that the condition with a vertical distractor produced more false alarms than all other conditions, all *t*(52) > 2.03, all *p* < 0.047, all *d* > 0.27, but none of the other three conditions differed from one another, all *t*(52) < 1.08, all *p* > 0.27, all *d* < 0.15.

#### Reaction time

**Fig. 5:**
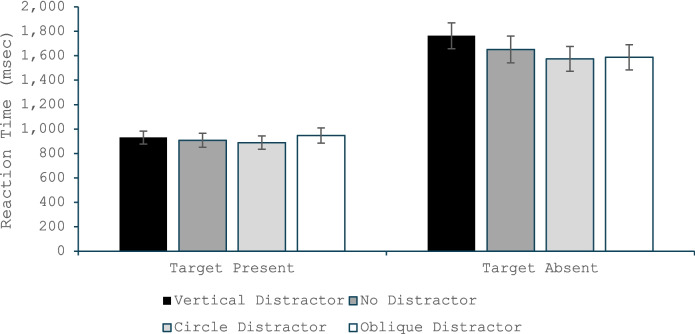
Reaction time in Experiment 2 as a function of whether the trial was a target present or target absent trial and whether the salient distractor matched the target’s orientation, did not appear, was a circle, or matched the common distractors’ orientation. Error bars are the standard error of the mean

The omnibus ANOVA (Fig. 5) found a main effect of target presence, *F*(1, 52) = 123.32, *p* < 0.001, η_p_^2^ = 0.70, with slower RTs for the target-absent than target present trials. There was also a main effect of distractor type, *F*(3, 156) = 10.69, *p* < 0.001, η_p_^2^ = 0.17, and a Target Presence × Distractor Type interaction, *F*(3, 156) = 9.42, *p* < 0.001, η_p_^2^ = 0.15. The ANOVA on only the target-present trials revealed no significant effect of distractor type, *F*(3, 156) = 1.31, *p* = 0.27, η_p_^2^ = 0.03. The ANOVA on target-absent trials found a significant difference across distractor types, *F*(3, 156) = 24.28, *p* < 0.001, η_p_^2^ = 0.32, and follow-up paired *t* test revealed that target-absent responses were made more quickly in the oblique distractor condition, *t*(52) = 3.01, *p* = 0.004, *d* = 0.41, and in the circular-distractor condition, *t*(52) = 2.99, *p* = 0.004, *d* = 0.41, than the no-distractor condition. However, target-absent responses took longer in the vertical-distractor condition than the no-distractor condition, *t*(52) = 3.80, *p* < 0.001, *d* = 0.41. The circular- and oblique-distractor conditions did not significantly differ, *t*(52) = 0.70, *p* = 0.49, *d* = 0.10.

### Discussion

Experiment 2 replicates the key findings of Experiment 1. We found the traditional QTE effect (more misses and faster target-absent RTs) when the salient distractor’s orientation matched the orientation of the common distractors, but this pattern did not occur when the salient distractor’s orientation matched the orientation of the target. Like Experiment 1, the effect flipped for target-absent RTs, but unlike in Experiment 1, in this experiment the vertical distractor did not result in more hits than the no-distractor condition. Further, a circular distractor, a distractor that has features not associated with the typical target-absent or target-present response, produces the standard QTE effect and behaves fairly similar to a distractor whose orientation matches the common distractor. The fact that the circular distractor, a distractor whose features are equally associated with both a target present and target-absent response, produces the standard QTE seems to rule out an explanation of the QTE finding on the basis of some type of late-stage response competition.

## General discussion

Across both experiments, we replicated the original QTE finding that a salient distractor that matches the orientation of the common distractors results in increased misses and faster target-absent RTs. In Experiment 2, a circular distractor had a similar effect, suggesting that the effect does not depend critically on the match between the salient distractor’s feature and the features of the common distractors. However, in both experiments this pattern reversed when the salient distractor shared features with the target—it produced slower target-absent RTs than displays without a salient distractor in both experiments, and fewer misses in Experiment 1. This pattern suggests that not all salient distractors lower quitting thresholds, which calls into question the QTE nomenclature and raises additional questions. Specifically, why does the effect flip when the distractor matches the features of the target? In addition, why should the salient distractor influence RTs and not accuracy in the target-absent trials, but accuracy and not RTs in the target-present trials?

**Fig. 6:**
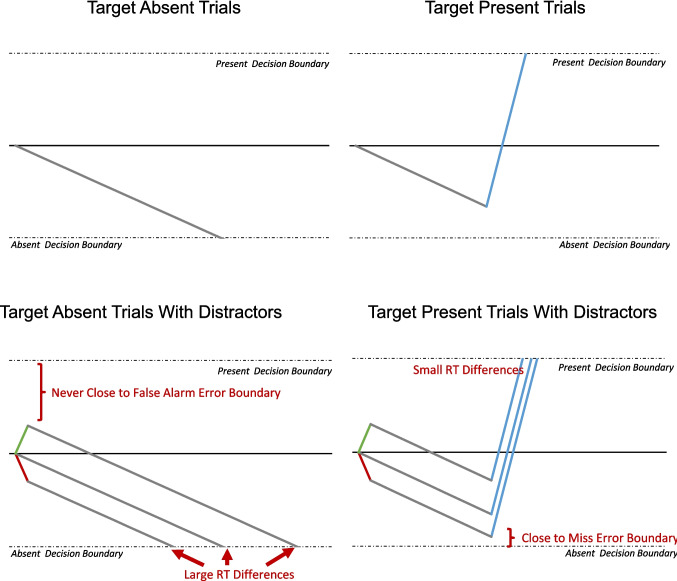
A cartoon of the drift diffusion process for visual search. The top two panels show the model for target absent (left) and present (right) trials with no distractors. The downward sloping gray line represents the slow accumulation of evidence for a “target absent” response that occurs as distractors are sampled. The steep blue line represents rapid accumulation of evidence for a “target present” response that occurs once the target is selected. The bottom panels depict the same scenarios where there are salient distractors. The green line represents the rapid bias toward a target present decision caused by a salient distractor that matches the target feature and the red line depicts the rapid bias toward an “absent” response caused by the distractor that does not match the target features. Note that for target absent trials the model predicts larger RT effects but limited accuracy effects. For target present trials it predicts robust accuracy effects but minimal RT effects. We note that in this cartoon the lines depict the average rate of accumulation, but given the noisy way information is accumulated, there would be substantial fluctuations from this mean drift rate

To answer these questions, we invoke the logic of a drift diffusion decision model but apply it to visual search. To understand this approach, we begin by considering search displays without a salient distractor. According to this view, as one samples more of the display, evidence accumulates (noisily) toward one of two decision boundaries, a target-present boundary and a target-absent boundary (see Fig. 6, top panels). However, given the abundance of distractors in a typical visual search task when one begins to sample items, they are most likely to be distractors, at least for inefficient search tasks (those with limited guidance). Thus, evidence will start to accumulate slowly toward the target-absent boundary. We say slowly because identifying any one distractor is not diagnostic of whether the target is present or not; one must sample many distractors to start to have confidence that the target is not present. Indeed, in target-absent trials, each new sampled item is a distractor so evidence slowly accumulates toward the distractor target boundary, and usually eventually reaches it.

For target-present trials, the process begins similarly. Even though there is a target present, most of the items in the display are distractors, so initially it is likely that people will sample distractors and slowly accumulate evidence toward the target-absent boundary. On some trials, the observer will continue to sample distractors and the slow accumulation of distractor evidence will hit the target-absent boundary prior to selecting the target, resulting in a miss. However, on many trials the observer will eventually sample the target (on average, halfway through the display if the display provides for little search guidance). The target is extremely diagnostic of the correct response; thus, once it is selected evidence accumulates rapidly toward the target present boundary.

Under this model, a shift in quitting thresholds would involve shifting the target-absent boundary toward the starting point, such that it would require less evidence to reach the decision boundary. That type of shift would predict the traditional QTE effect—misses would be more likely to occur because the absent boundary would be easier to reach and target-absent RTs would be faster because one would need to accumulate less evidence before responding target absent. That model should not impact false-alarm rates, since the distance from the starting point to the target-present boundary would be unaffected, but the model would predict no change in target-present RTs, something that is occasionally but not always found.

However, there are two problems with this model. First, generally in these models the decision boundaries are set by global task parameters and do not change mid trial, which would be required to explain the QTE—after seeing the distractor one would rapidly adjust the criteria for that particular trial. Second, the model cannot easily handle our current finding that a salient distractor that matches the target reverses the effect. To accommodate that result, one would have to posit a system where the presence of most salient distractors would create a shift in the target-absent boundary, but the salient distractor that shares features with the target would create a shift in the target-present boundary—and again both of these would be mid trial.

However, we believe there is a more parsimonious resolution that does not suffer from these issues. Briefly, due to its high salience, the distractor leads to an early, brief period of rapid accumulation of information toward its associated boundary. If it is a distractor object, with features matching the common distractor or novel features, this results in an initial rapid accumulation of evidence toward the target-absent boundary, and then search proceeds as normal. If it has target features, there is an initial rapid accumulation toward the target-present boundary and then search proceeds as normal. In short, because of its salience the distractor acts like a super-stimulus in terms of evidence accumulation.

This model can account for the patterns we have found in these experiments (See Fig. 6, bottom panels). It accounts for the faster target-absent RTs with oblique and circular distractors; in both these cases, the initial burst of evidence toward the target-absent boundary leads to reaching that boundary sooner. The model also accounts for the increase in misses with a distractor that does not share its orientation with the target, because even in target-present trials the initial drift, as one samples distractors, is toward the target-absent boundary and the additional burst of evidence in that direction leads to more opportunities to reach the target-absent boundary before the target is selected for scrutiny.

The model also readily handles the reversal seen when the salient distractor has features that match the target. In this case, the initial burst of activity is toward the target present boundary. In target-absent trials, after this initial burst, the observer samples distractors and evidence is accumulated toward the target-absent boundary, so the initial burst is unlikely to produce a false alarm but is likely to slow target-absent RTs. We also think it noteworthy that the model accounts for the finding that the effects are typically isolated to the accuracy data for target-present trials, and isolated to RTs for target-absent trials.

While our conceptual model appears to account for the patterns in our data, we sought to quantitatively verify whether this type of model would indeed produce these patterns. To do so, we programmed a simulation in Python to implement this model for Experiment 1 (the annotated code for the model that can be run and manipulated is available OSF at https://osf.io/qawvt/).

The simulation involves running a drift diffusion model, where the accumulated evidence toward one of two decision boundaries (present/absent) updates recurrently. Each iteration updates the accumulated evidence by adding the mean drift rate plus a noise parameter selected from a Gaussian distribution. In the model, the mean drift rate is initially set to a slow accumulation toward the target-absent boundary to simulate the sampling of relatively uninformative distractors. This mean drift rate changes to a steep drift toward the target-present boundary if and when the target is located.

On each "trial," the model determines when the target is located based on a Gaussian distribution with a relatively large standard deviation, reflecting the variability in human behavior—sometimes people scrutinize the target early in the display, and sometimes later. The salient distractors are implemented as an initial accumulation of evidence toward the boundary associated with the orientation of the distractor—toward the target-absent boundary for the oblique-salient distractor and toward the target-present boundary for the vertical-salient distractor.

While the precise values of the model parameters are somewhat arbitrary, we set the decision boundaries to be 100 units away from the starting point of the drift model. The mean drift rate while sampling distractors was two units per iteration toward the absent boundary, with additional error drawn from a Gaussian distribution with a mean of zero and a standard deviation of 10. The model iteratively updated the evidence in this manner until either a decision boundary was crossed or, for target-present trials, when the model “found the target” and began accumulating evidence for the target. At this point, the drift rate reversed sign, and the mean drift rate accelerated to 10 units per iteration, plus the same type of error noted above.

**Fig. 7:**
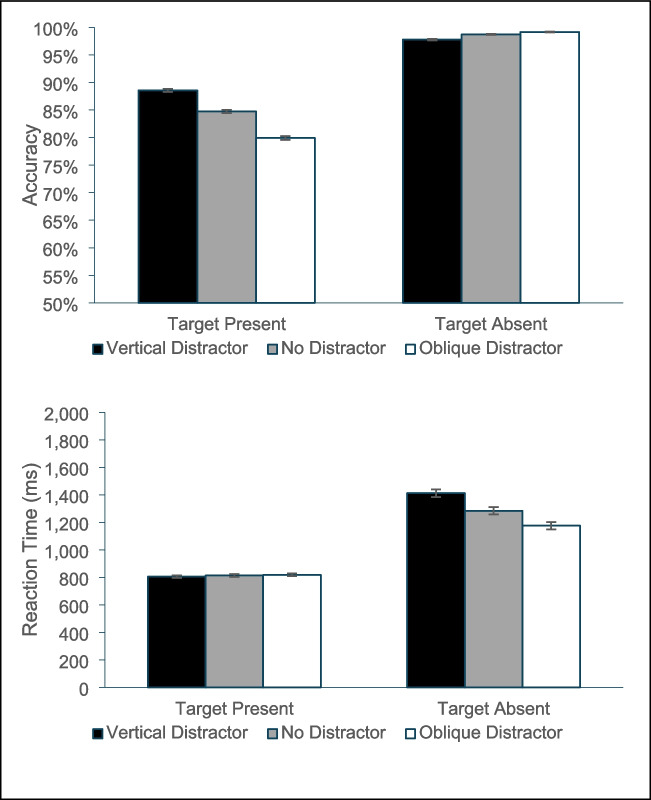
Accuracy (top panel) and reaction time (bottom panel) data from the quantitative simulation of our proposed model. Data are based on a simulation of a 500 participant experiment. The patterns in the data are very similar to those from Experiment 1

For each target-present trial, the iteration at which the model found the target and switched its mean drift rate was drawn from a Gaussian distribution with a mean of 20 iterations and a standard deviation of 10. Finally, to convert model iterations to RT, we assumed that each iteration corresponded to 25 ms. We then simulated the experiment with 40 subjects and later with 500 subjects. The results from both simulations were highly similar to each other and closely matched our observed patterns of findings. Figure 7 plots the data from the simulation of 500 subjects.

The fact that this computational implementation of our conceptual model produces similar patterns of results—including the reversal in effects with the change from a vertical- to an oblique-salient distractor—and that most of the effect is reflected in RT for target-absent trials and accuracy for target-present trials, provides additional support for this type of model.

**Fig. 8:**
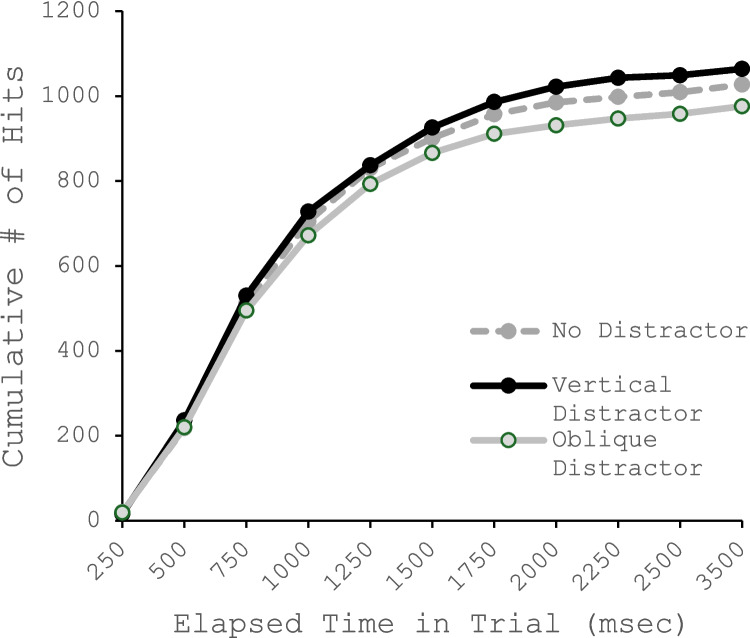
Cumulative number of hits in Experiment 1 as function of elapsed time in the trial and distractor type

Even so, it is worth noting that there was an inconsistency across our two experiments concerning the effect that a vertical salient distractor had on target-present accuracy. In Experiment 1, this type of distractor increased target detection, whereas it did not for the second experiment. Although this finding is not robust, and thus we do not wish to make too much of it, we can imagine three ways that a salient distractor that matched the orientation of the target could increase target-present detections. First, it is possible that its presence leads to rapid target-present responses based on the distractor. Second it is possible that the initial burst of activity toward the target-present boundary resulted in fewer identification misses—misses where the target is fixated but misidentified as a distractor. Third, given that the vertical distractor produced longer target-absent RTs, its presence may have reduced the likelihood of a miss due to a search error—never fixating the target before responding target absent. In Experiment 1, we have no evidence for the first explanation; there were no more false alarms in that condition (although there were in Experiment 2) and an inspection of the cumulative number of target present responses as function of elapsed search time shows no evidence of increased target-present responses early in the trial (see Fig. 8). Eye tracking would help differentiate between the latter two alternatives.

Finally, we note that, unlike the original report (Moher, 2020), we did not find that the salient distractor slowed RTs for target-present hits. We also note that the findings of faster target-absent RTs and increased misses are replicated in most reports of the QTE, and our model suggests these two effects should be large. By contrast, the slowing of target-present RTs is found in some, but not all, prior reports of the QTE (e.g., Lawrence & Pratt, 2022). This variation might be expected, as our model suggests that while there should be an effect, it should be relatively small, making it difficult to detect.

While speculative, our model also suggests that the magnitude of target-present slowing due to an oblique distractor should increase as the slope of the drift rate associated with inspecting a target becomes shallower. Indeed, if one imagines that the blue lines in our cartoon were shallower, the points where the conditions would reach the target-present boundary would spread apart, representing a difference in RTs. Additionally, our computational model verified that reducing the drift rate associated with detecting the target produced target-present slowing for an oblique distractor. Thus, some visual factors, such as the size of the target or its low-level saliency, may impact the accrual rate of information when the target is inspected, leading to instances where this effect is more pronounced.

Similarly, in our computational model, we initially assumed that the distractor would have an impact on every trial when present. This assumption was based on its saliency (due to its unique color, size, and onset), which should readily capture attention and influence search. However, to the extent that the distractor does not reliably capture attention, its impact should be diminished. Indeed, when we adjusted our model so that the initial bias in information occurred only on a subset of trials, the distractor effects diminished accordingly. This need for the distractor to initially attract attention in order to be influential may explain why some studies have failed to find the QTE with less salient stimuli. For instance, Lawrence and Pratt (2022) found the QTE with large but not small distractors. The larger stimuli were presumably more unique and salient, making them more likely to impact search on a greater proportion of trials.

Furthermore, it is worth noting that we might have expected minimal capture by the salient distractor in our design. In most QTE experiments, the target was a single vertical line among two types of oblique distractors that were mirror images of each other. Given that mirror images are difficult to discriminate (Gregory & McCloskey, 2010), the targets in these displays may have been perceived as an orientation singleton, meaning participants might adopt a singleton search mode (Bacon & Egeth, 1994; Lamy & Egeth, 2003), which would make capture by other singletons, such as the salient distractors, more likely. However, in our design, the addition of the vertical distractor made the search less of a singleton search and more of a conjunction search, which may have pushed participants into feature search mode—a mode less prone to capture by irrelevant singleton stimuli (Bacon & Egeth, 1994). Despite this, we still found fairly robust effects of the distractor, suggesting that it was capturing attention and influencing search. However, these salient stimuli had multiple factors contributing to their saliency: they were a unique size, a unique color, and had unique onset times. These multiple unique properties—and perhaps, most importantly, the unique onset times (Franconeri & Simons, 2003; Lamy & Egeth, 2003; Yantis & Jonides, 1984)—may have been critical in ensuring that these stimuli continued to attract attention in our design.

## Conclusions

Across two experiments we replicate the main finding of the QTE, that a salient distractor increases target misses and decreases target-absent RTs. However, we show that this effect reverses, when the relevant features of the distractor match the target. That reversal suggests that not all salient distractors produce a reduction in search-quitting thresholds, and our interpretation is that the effect is not to reduce the amount of evidence required to make a target-absent decision but instead is that the distractor, due to its salience, produces a brief period early in the trial when evidence is rapidly accumulated toward the boundary most closely associated with the distractor’s relevant features. Thus, distractors that share no features with the target produce rapid accumulation toward the target-absent decision threshold, thereby producing fast absent RTs and more misses. By contrast, distractors that share features with the target produce an initial burst of evidence accumulation toward the target-present boundary, thereby slowing RTs in target-absent trials, and reducing misses in target present trials.

## Data Availability

Processed subject level data is provided on OSF at https://osf.io/qawvt/.

## Footnotes

1 The one exception to this we found was that Moher (2020) had a *T* among *L* search in Experiment 3. However, the salient distractor was always an *L*, so it still matched the common distractors.

2 We acknowledge that this is a high number of noncompliant subjects. The online experiment was run on 2 days—a Friday and a Tuesday. Over 70% of the noncompliant subjects (19 of the 27) were run on Friday. While this might be coincidental, we will not be running online experiments over the weekend again.

## Code availability

The E-Prime experiment build files and stimuli are available: https://osf.io/qawvt/

## Declarations

### Open practices statement

The experiments were not preregistered, but the data, stimuli, and experimental files are publicly available: https://osf.io/qawvt/
